# Supplementary material for: GTExome: Modeling commonly expressed missense mutations in the human genome
Source: PLoS One. 2024 May 30;19(5):e0303604. doi: 10.1371/journal.pone.0303604 (PMC11139294; doi:10.1371/journal.pone.0303604)
Supplement: S1 File — (DOCX) [file pone.0303604.s001.docx]

**Supporting Information for:**

**GTExome: Modeling commonly expressed missense mutations in the human genome**

Jill Hoffman, Henry Tan, Clara Sandoval-Cooper, Kaelyn de Villiers, Scott M. Reed

**Table S1.** Matched genes to known adverse drug reactions (from Swen 2023).


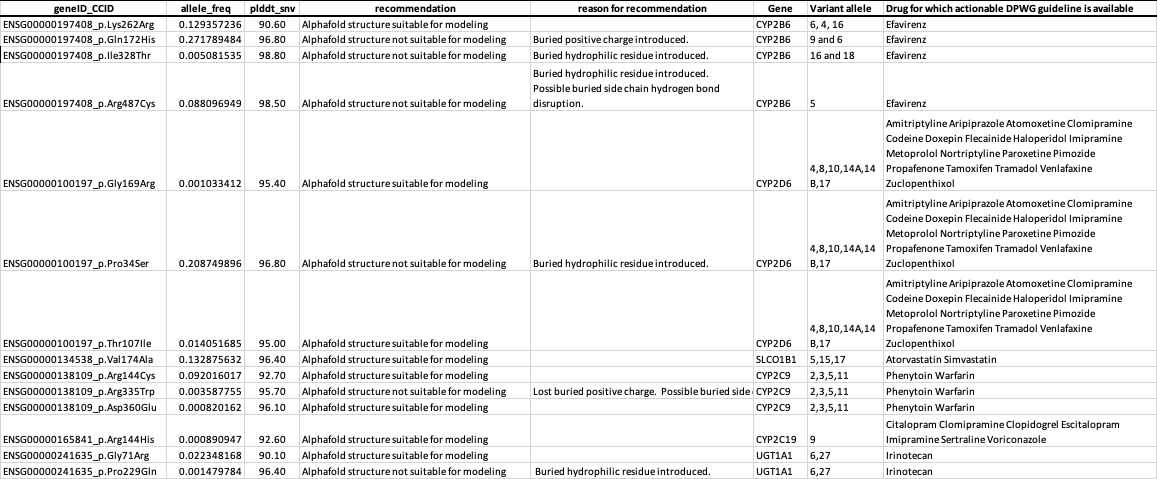


**Table S2.** Protein length and RMSD values for structures compared to ColabFold (image overlays below).

**Table S3.** Protein length and RMSD values for experimental structures analyzed.


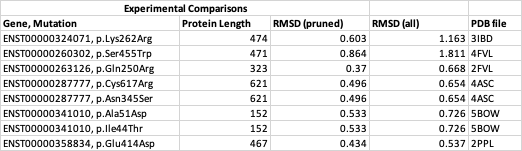


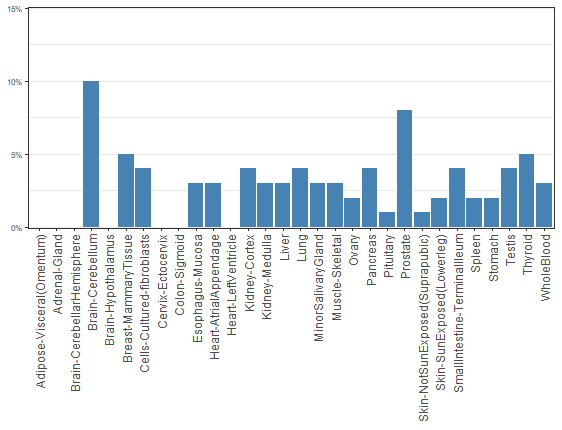


**Figure S1.** Percentage of missense SNVs involving positive to negative or negative to positive residue swap for residue by tissue type (for tissues in GTEx with > 10 SNVs).


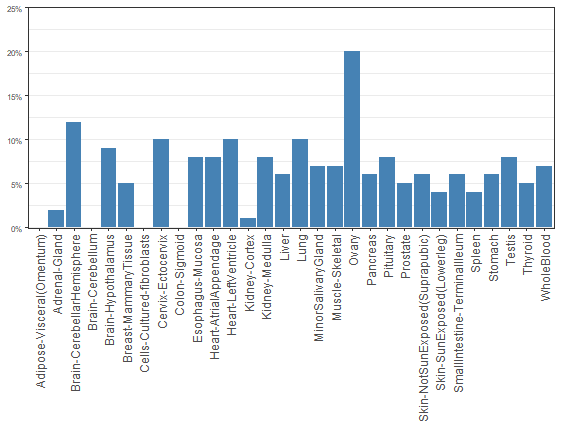


**Figure S2.** Percentage of missense SNVs involving loss of a hydrogen bond by tissue type (for tissues in GTEx with > 10 SNVs).


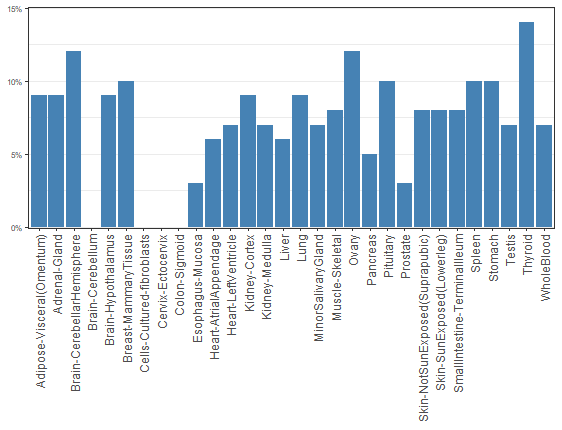


**Figure S3.** Percentage of missense SNVs involving loss of proline with a cis configuration by tissue type (for tissues in GTEx with > 10 SNVs).


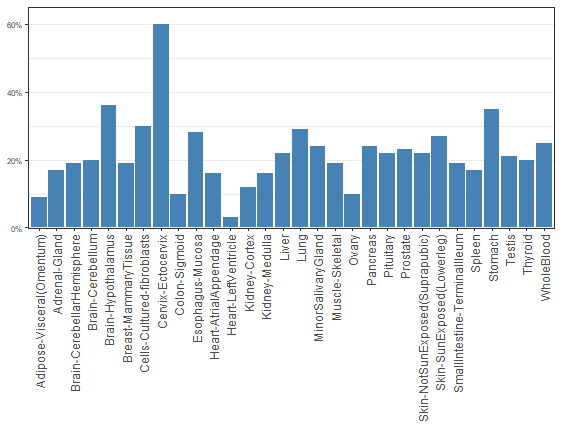


**Figure S4.** Percentage of rare (allele frequency <0.01) SNVs by tissue type (for tissues in GTEx with > 10 SNVs).


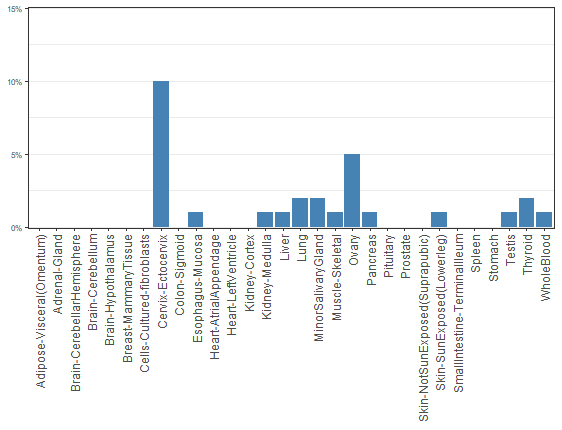


**Figure S5.** Percentage of missense SNVs involving loss of a buries salt bridge by tissue type (for tissues in GTEx with > 10 SNVs).

**Method for Figure S6 (Proteins 1 – 47):** Chimera Matchmaker overlay of ColabFold produced structures containing missense mutation with GTExome produced structure where side chain was repacked at the SNV and within a given using FASPR. For each radius, R, a GTExome structure was produced. Each residue of that repacked mutated file was then compared to the analog residue in the native protein file for each repacked radius and the distance was calculated on each atom in the side chain:

$$vector\_difference= \left| coords_{atom1}-coords_{atom2} \right|$$

$$distance= \sqrt{sum(vector\_difference^{2})}$$

If the distance calculated on any atom was larger than 0, the residue counted as being adjusted (count shown on y-axis of each graph as a function of radius). To account for the possibility of symmetrical residues (Phe, Tyr, Leu, Val) coordinates being flipped, the flipped and un-flipped residues were evaluated. If the distance of the un-flipped case was larger than the flipped case, the residue was determined to have been flipped. Whichever case that was determined was used in the overall distance calculation to determine if it was repacked.

Images of overlay shown with 30 Å radius.

Protein 1

Protein 2

Protein 3

Protein 4

Protein 5

Protein 6

Protein 7

Protein 8

Protein 9

Protein 10

Protein 11

Protein 12

Protein 13

Protein 14

Protein 15

Protein 16

Protein 17

Protein 18

Protein 19

Protein 20

Protein 21

Protein 22

Protein 23

Protein 24

Protein 25

Protein 26

Protein 27

Protein 28

Protein 29

Protein 30

Protein 31

Protein 32

Protein 33

Protein 34

Protein 35

Protein 36

Protein 37

Protein 38

Protein 39

Protein 40

Protein 41

Protein 42

Protein 43

Protein 44

Protein 45

Protein 46

Protein 47
