# Supplementary material for: GTExome: Modeling commonly expressed missense mutations in the human genome
Source: PLoS One. 2024 May 30;19(5):e0303604. doi: 10.1371/journal.pone.0303604 (PMC11139294; doi:10.1371/journal.pone.0303604)
Supplement: Attachment — Submitted filename: Response to Reviewers.docx [file pone.0303604.s002.docx]

**Response to Reviewers**

**Reviewer #1:** Hoffman et al. describe a web resource that aims to simplify the analysis of the impact of missense mutations on structure for human proteins. Their focus is on 9,666 common mutations from gnomAD database. Where experimental structures are not available for modeling mutations, they use AlphaFold2 models.

Major Points:

It is unclear how this resource differs from or improves on Missense3D? It appears this work is on a smaller set of under 10k mutations while Missense3D has over 4 million modeled mutations. It also describes similar structural effects. It is possible that GTExome is more accurate, but a proper benchmark and comparison is required to show that.

There is overlap with Missense3D because similar criteria are used to flag missense mutations that may be deleterious. The biggest difference between the two resources is that missense3D-DB produces a text file listing conclusions about the mutation. GTExome in contrast, produces 3D coordinates for that protein allows you to visualize the protein and then allows you to download the 3D structure. Missense3D which previously required a PDB structure recently added the note “You can now use AlphaFold models in Missense3D, just upload your AlphaFold model using the 'Position on 3D structure' mode.” While this is now an option, it requires finding the suitable AlphaFold model and uploading it. The missense3D-DB is presumably still based on their pre-AlphaFold Phyre based structures. In contrast, GTExome stores all the AlphaFold structures locally, produces the mutant structures on the backend instantly and presents them to the user. One could produce similar structures by downloading structure files and then uploading them to Missense3D. One would need to submit each job individually and wait for email notification that the job was complete. GTExome also provides the user flexibility in setting the radius of repacking allowing a user to preserve side-chain coordinates as needed. Furthermore, GTExome provides a sorting method through the gtex input tab which is central to the primary purpose of the tool. Simply generating structures is not the primary purpose of the tool.

It is unclear why the authors are focusing on the set of 9,666 missense mutations. They point out their use of AlphaMissense to globally analyze their set but only find 6% are pathogenic. This is likely because they are only using mutations common in the population which are likely to be benign. I think the resource would have a larger impact if it focused on pathogenic variants.

The tool is not suitable exclusively for the 9,666 mutations that were highlighted. A user could use this tool to examine exclusively pathogenic variants. We agree this would be interesting and that the contrast in properties between pathogenic and non-pathogenic variants might be instructive as to the evolutionary pressure on these sites. We anticipate that we will explore this in the future. A specific benefit of our initial study of 9,666 mutations is exactly as the reviewer stated that there will be a bias toward selecting benign mutations. In studying adverse drug reactions, these are the mutations that are more likely to be present in populations. Many pathogenic mutations may not persist because the protein is unstable or because the protein is essential and its misfunction results in non-viability. In contrast, the mutations in our sample study are more likely to be explanatory for adverse drug reactions and we added a sentence to the manuscript explaining this.

Additionally, the authors compare their mutation set to a list of mutations that cause adverse drug reactions and find an overlap of only 14 mutations across 6 genes. This does not appear to be enriched and is likely just based on random chance. A statistical test should be used to determine in the overlap is significant.

The paper describing these mutations “A 12-gene pharmacogenetic panel to prevent adverse drug reactions: an open-label, multicentre, controlled, cluster-randomised crossover implementation study” examined a small number (12) of genes. Finding overlap with 6 of these genes is a substantial fraction considering that there are 71 million possible mutations. However, we did not make the argument in the manuscript that this subset of missense mutations is enriched in mutations that have ADR-explanatory power. Our set includes genes that are 1) highly expressed in one tissue type over all others but excludes proteins that are highly expressing across multiple tissue types and 2) that have allele frequencies greater than 0.01. We have added text explaining point 2 in the manuscript which we failed to include previously. Furthermore, we think the apparent enrichment is likely the result of the second fact not the first as there is no reason to expect an ADR-explaining mutation would occur in a protein that expresses highly in one tissue. To disentangle and test whether there was enrichment due to either reason it would be necessary to create a similar size sample of mutations with similar allele frequencies that were not selected based on tissue type. This would likely be overinterpreting the data as this panel of 12 genes are likely a small subset of all genes that can explain adverse drug reactions. We have clarified the text, adding the following “The overlap of this study set with 6 of these genes is likely the result of our focus on genes that have allele frequencies greater than 0.01 not the tissue specificity although it is important to note that levels of genes expression do not translate directly to level of protein.”

I found the website was not intuitive to use. I followed the instructions by selecting the tissue type and clicking submit but nothing happened. I also used the ‘exac’ tab and entered a gene of interest, clicked a mutation but when I attempted to model it, the website stalled “searching for AlphaFold2 Structure”.

We have added a more specific set of instructions to the GTExome site including instructions on how each tab can be used behind an info button on the top of the page. Furthermore, we have added placeholder text in each of the input boxes. This gives the user a sample to guide them and helps avoid problems like accidentally using a transcript ID instead of a gene ID. Finally, we added a walkthrough video that covers the three entry points with examples. There are cases where if a user makes changes to some data such as the geneID and includes a non-functional geneID they get no response. We are working on a solution for this and for now have clarified how to change the geneID in the newly added instructions and walk-through video. In all other cases, such as a non-existent AlphaFold structures a specific warning is provided.

Minor Points:

Figure 1 is difficult to interpret. The text is hard to read on some panels and the resolution is low.

We have re-done the figure improving the resolution and clarity.

**Reviewer #2:** The authors present a useful web-server that allows a range of analyses and bring together a range of resources such GTEx and AF. The whole idea, in my understanding, is to provide a platform to analyze point mutations. Users can select their tx of interest, look at expression and map mutations iwth the added bonus of been able to visualize the impact of the mutation at structural level. Needless to say that this approach have some limitations, which are not to blame to the work done by the authors. For instance, levels of genes expression do not translate directly to level of protein, e.g. high gene expression not equal to high levels of protein as there are regulatory mechanisms beyond transcription. Mutations not only affect the structure of proteins, could also affect post-translation modifications that can equally be very important for the function of the protein. AF was not designed to assess the impact of mutations although some publications have shown
that structural model containing mutations predicted by AF could provide usulf information. Finally, mutations could not only cause structural changes at local level, but also at global on, or cause problems with interactions with other proteins and or natural partners. These two  are indeed not covered. Understand that the authors show a few cases where can be seen that structural models of mutants and same done experimentally do agree. Authors would have also to agree that this is not a systematic analysis

We agree that this is not a comprehensive analysis. Time constraints require selecting a subset of mutations and we took one approach. Others may prefer a different approach and our tool enables others to explore different subsets. We agree with all the caveats mentioned and added the following point to the manuscript “it is important to note that levels of genes expression do not translate directly to level of protein.”

Regarding the usage of the web-server; the searches were fast and responsive, although the actually modelling of mutants did not return any results in two cases I tried (Mon 18th), so I cannot comment in this aspect.

We hope the changes mentioned above help increase the chances of users getting the output they expect.
