# Supplementary material for: GTExome: Modeling commonly expressed missense mutations in the human genome
Source: PLoS One. 2024 May 30;19(5):e0303604. doi: 10.1371/journal.pone.0303604 (PMC11139294; doi:10.1371/journal.pone.0303604)
Supplement: Attachment — Submitted filename: Response to Reviewers.docx [file pone.0303604.s003.docx]

**Subject:** Response to Reviewer Comments - Revised Submission of Manuscript PONE-D-24-03381

Dear Dr. Yong Wang,

We are grateful for the opportunity to resubmit our manuscript, "GTExome: Modeling Commonly Expressed Missense Mutations in the Human Genome," to PLOS ONE. We appreciate the thorough review and constructive comments provided by the academic editor and the reviewer. We have carefully addressed all the points raised and believe these changes have significantly improved our manuscript.

As requested, we have uploaded three separate files: a rebuttal letter (this document), a marked-up copy of our manuscript highlighting the changes made (Revised Manuscript with Track Changes), and an unmarked version of the revised paper (Manuscript).

Below, we summarize how we addressed the major points in your request and the reviewer's comments:

1. **PLOS ONE's Style and File Naming Requirements:** We have revised our manuscript to meet the style requirements as outlined and have ensured all files are correctly named according to the provided templates.
2. **Code Sharing Guidelines Compliance:** In line with PLOS ONE's code sharing policy, we have made all author-generated code available without restrictions.
3. **Financial Disclosure and Funder's Role:** We have amended our financial disclosure to fully spell out "National Institutes of Health".
4. **Reference to Figure 3 and Supporting Information Captions:** We have ensured that Figure 3 is referred to appropriately within the text and have ensured all figures have captions in the Supporting Information files as instructed.
5. **Reference List Accuracy:** The reference list has been thoroughly reviewed and updated to ensure completeness and correctness.

Addressing Reviewer's Comments:

1. The word "feasible" modifying missense mutations has been removed as unnecessary and confusing.
2. We have corrected our COSMIS analysis and changed the text to reflect our updated evaluation to say, “… median COSMIS value for the 3493 mutations of this study set that were in the COSMIS database was 0.3882 for values derived from AlphaFold structures and 0.4819 for PDB derived structures.” This revised statement clarifies we are reporting COSMIS values not COSMIS p-values and that we re reporting median values. The conclusion remains unchanged.
3. PDB codes for structures determined by x-ray crystallography have been provided as requested in table S3.
4. A correction has been made to the location of the citation of Figure 2 within the paragraph.
5. The sentence mentioning Dynamut software has been trimmed and the reference to this software removed as it did not add to the clarity of the manuscript.
6. The confusion between proteins and mutations discussed on pages 8 and 17 has been clarified, with specific proteins explicitly mentioned.

We trust that these revisions address the concerns raised during the review process. We thank you again for the opportunity to improve our manuscript and for considering it for publication in PLOS ONE.

Kind regards,

Prof. Scott Reed
Department of Chemistry
University of Colorado Denver
1151 Arapahoe St.

Science Building 4131
Denver, CO 80217-3364
[scott.reed@ucdenver.edu](mailto:scott.reed@ucdenver.edu)

(303) 315-7634
